# A Study of Disease Prognosis in Lung Adenocarcinoma Using Single-Cell Decomposition and Immune Signature Analysis

**DOI:** 10.3390/cancers16183207

**Published:** 2024-09-20

**Authors:** Cheng-Yang Lee, Yu-Chung Wu, Tze-Chi Liao, Shih-Hsin Hsiao, Justin Bo-Kai Hsu, Tzu-Hao Chang

**Affiliations:** 1 Graduate Institute of Biomedical Informatics, College of Medical Science and Technology, Taipei Medical University, Taipei 110301, Taiwan; nathanlee@tmu.edu.tw (C.-Y.L.); m610109004@tmu.edu.tw (T.-C.L.); 2 Division of Thoracic Surgery, Department of Surgery, Taipei Medical University Hospital, Taipei 110301, Taiwan; yuchungwu@tmu.edu.tw; 3 Department of Surgery, Division of Thoracic Surgery, School of Medicine, College of Medicine, Taipei Medical University, Taipei 110301, Taiwan; 4 Division of Pulmonary Medicine, Department of Internal Medicine, School of Medicine, College of Medicine, Taipei Medical University, Taipei 110301, Taiwan; hsiaomd@tmu.edu.tw; 5 Division of Pulmonary Medicine, Department of Internal Medicine, Taipei Medical University Hospital, Taipei 110301, Taiwan; 6 Department of Computer Science and Engineering, Yuan Ze University, Taoyuan 320315, Taiwan

**Keywords:** lung adenocarcinoma, bulk RNA sequencing data decomposition, immune signatures, disease prognosis

## Abstract

**Simple Summary:**

The tumor microenvironment (TME) influences treatment outcome, and analysis of immune cell composition plays an important role in establishing effective prognostic models. This study investigated cellular proportions decomposed from Rulk RNA expression data and immune profiles of patients with lung adenocarcinoma (LUAD) using publicly available data from TCGA and GEO. The results of the study showed a correlation between specific immune signatures, poor prognostic signatures (PPS) and patient outcomes such as progression-free survival and chemotherapy response. We integrated these features and used machine learning models to predict prognosis, with support vector machines (SVMs) having the highest accuracy. This study highlights the importance of immune profiling in advancing precision medicine for lung cancer patients.

**Abstract:**

**Background:** The development of tumors is a highly complex process that entails numerous interactions and intricate relationships between the host immune system and cancer cells. It has been demonstrated in studies that the treatment response of patients can be correlated with the tumor microenvironment (TME). Consequently, the examination of diverse immune profiles within the TME can facilitate the elucidation of tumor development and the development of advantageous models for diagnoses and prognoses. **Methods:** In this study, we utilized a single-cell decomposition method to analyze the relationships between cell proportions and immune signatures in lung adenocarcinoma (LUAD) patients. **Results:** Our findings indicate that specific immune cell populations and immune signatures are significantly associated with patient prognosis. By identifying poor prognosis signatures (PPS), we reveal the critical role of immune profiles and cellular composition in disease outcomes, emphasizing their diagnostic potential for predicting patient prognosis. **Conclusions:** This study highlights the importance of immune signatures and cellular composition, which may serve as valuable biomarkers for disease prognosis in LUAD patients.

## 1. Introduction

Non-small-cell lung carcinoma (NSCLC) is the predominant form of lung cancer, accounting for more than 85% of cases [1]. NSCLC can be further categorized into subtypes, such as lung adenocarcinoma (LUAD) and lung squamous cell carcinoma (LUSC), based on histological features [2,3]. Treatment options for NSCLC include surgery, radiation therapy, chemotherapy, immunotherapy, and targeted therapy, often used in combination. Immunotherapy utilizing checkpoint inhibitors for programmed death 1 (PD-1) and programmed death ligand 1 (PD-L1) has revolutionized cancer treatment and is now employed as first-line or second-line therapy for lung cancer. However, only a small percentage of patients have derived significant benefits from immunotherapy [4]. Chemotherapy remains a fundamental approach in lung cancer treatment, with 53.4% of stage 1 NSCLC patients undergoing chemotherapy [5].

It is becoming increasingly clear that tumor development is a complex process that involves a number of different interactions between cancer cells and the host immune system. The tumor microenvironment (TME) is understood to play an important role in determining the response to chemotherapy, with an efficient immune system shown to optimize chemotherapeutic effects [6]. Given the complexity of the TME, it is becoming increasingly clear that a combination of biomarkers is required to predict individual responses to treatment. The concept of immunograms has been introduced as a potential tool to assess the immune status of cancer patients visually [7]. Blank et al. developed cancer immunograms with the aim of representing interactions between the immune system and cancer cells, with the intention of aiding in biomarker research and clinical drug selection [8]. Additionally, tools like ESTIMATE estimate the content of tumor and immune cells to understand tumor purity [9]. Karasaki et al. used whole-exome and RNA sequencing on 20 NSCLC patients, creating radar plots that revealed three immune patterns: T-cell rich, intermediate, and T-cell depleted [10]. Immunograms are valuable biomarkers that provide insights into the TME, though there is a view that more T-cell-related factors could be incorporated. Furthermore, Donghai et al. validated immunological features in melanomas, showing that ImmunCell.Sig includes gene features of immune cell subtypes and can predict immunotherapy responses [11].

Single-cell sequencing is a recently developed and widely used technique that enables a more detailed exploration of cell types and compositions compared to bulk RNA-Seq. However, this kind of sequencing method is expensive. Several computational tools, such as Bisque, CIBERSORT, CIBERSORTx, MuSiC, and various algorithms, have been developed to estimate immune cell proportions using bulk RNA-Seq data [11,12,13,14]. Some researchers utilized cell-type decomposition to cluster lung cancer patients based on major components of the TME. Various publicly available data resources, such as The Cancer Genome Atlas (TCGA) for bulk RNA-Seq data and DISCO, GEO, and CancerSEA for single-cell (sc)RNA-Seq data, provide valuable information for cell type decomposition [15,16,17].

Previous studies have contributed to the development of several deconvolution techniques that have been used to estimate immune cell proportions. CIBERSORT [18], developed by Newman et al., employs 22 leukocyte signatures (LM22) as reference markers, though its accuracy may be limited in certain instances, potentially leading to errors in cell type estimation. In contrast, Bisque [13], introduced by Brandon et al. in 2020, has the potential to provide more accurate cell proportion estimates by combining bulk RNA and single-cell RNA sequencing (scRNA-seq) data. Chang Li et al. employed CIBERSORT to categorize NSCLC patients into subgroups based on tumor-infiltrating immune cells (TIICs), with the aim of identifying potential therapeutic targets [19]. Yang Zheng et al. applied non-negative matrix factorization (NMF) to categorize patients, revealing significant correlations between copy number alterations (CNAs) and immune checkpoint expressions [20]. This study aims to build on these insights by integrating immune profiles with single-cell decomposition techniques, with the goal of enhancing the diagnostic potential for predicting patient prognosis and improving precision medicine approaches for NSCLC patients.

## 2. Methods

### 2.1. System Flow and Datasets of the Study

**Figure 1:**
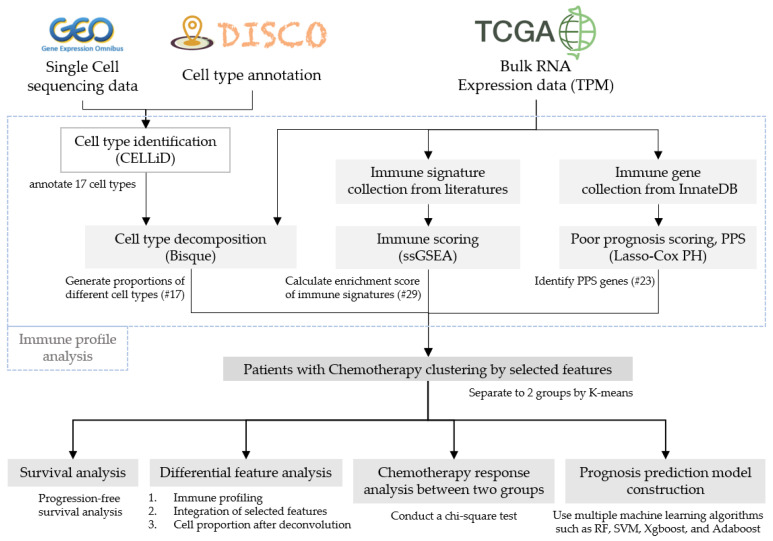
Research process flowchart.

This study can be briefly divided into three parts: an immune profile analysis of patients, immune clustering analysis, and prognosis model construction as shown in Figure 1, and as described in various subsections of “Section 2”. In addition, we utilized datasets including RNA-Seq data of bulk tumors with both raw read counts and transcripts per kilobase million (TPM) from 513 patients with LUAD in the TCGA database (https://portal.gdc.cancer.gov/, accessed on 24 January 2021), which corresponded to clinical and mutation data, including follow-up information, drug treatment, responses, and genetic alteration of ALK and EGFR. Of these patients, 177 had once received chemotherapy treatment. Also, we utilized scRNA-Seq data from 42 patients with NSCLC, which were used in Wu’s study [21] and can be downloaded from the Gene Expression Omnibus (GEO) database under the accession numbers GSE148071 (https://www.ncbi.nlm.nih.gov/geo/, assessed on 24 January 2021).

### 2.2. Immune Profile Estimations of Patients

In the beginning, we employed the CELLiD (cell-type identification) tool of the DISCO database for cell-type annotation, after several cell clusters were identified in compliance with the requirements of scRNA-Seq analytical tools. Meanwhile, 89,888 cells were identified in these scRNA-Seq data, for an average of 2140 cells per patient. The 17 different cell types identified among patients with LUAD (parts of 42 patients) included basal cells, classical dendritic cells (cDCs), differentiated ciliated cells, endothelial cells, epithelial cells, macrophages, mast cells, mesothelial cells, monocytes, myofibroblasts, plasma cells, proliferative epithelial cells, proliferative macrophages, secretory cells, smooth muscle cells, T cells, and type 2 alveolar epithelial cells. For the immune profile analysis, three processes were further performed. First, we used the BisqueRNA (R package, version v1.0.5) by importing single-cell sequencing data and combined those with bulk RNA read counts to analyze cell proportions of the 17 cell types in patients with LUAD. Second, we evaluated 29 immune signatures (Table S1) for each patient, which were integrated from 4 studies [7,10,11,22] and the ESTIMATES tool [9], using the single-sample gene set enrichment analysis (ssGSEA) scoring method. The ssGSEA method is extended from the GSEA method, which can be used to evaluate enriched levels (enrichment scores) of specific gene sets (existing gene signatures) in a single sample using expression data [23]. Finally, poor prognosis signature (PPS) genes were identified [19] based on immune-relevant genes, which were collected from the InnateDB database [24]. The Cox proportional hazards (CoxPH) model and the Lasso feature selection method of the R package were used to generate representative PPS genes. We set the parameter alpha of Lasso to 1 and lambda to 100. After that, 23 representative PPS genes were identified.

### 2.3. Immune Clustering Analysis

The K-means algorithm was employed to cluster patients into different groups based on their immune profiles. In this study, the parameter *k* was set to 2, and the patients were subsequently divided into two distinct groups (cluster 1 and cluster 2, designated as C1 and C2, respectively). Several different scenarios were additionally investigated between the two groups including survival durations, immunograms, and all kinds of features identified in the previous section, titled “Immune profile estimations of patients”. For survival durations, survminer (R package, version v0.4.9) was used to explore the progression-free interval (PFI) between the C1 and C2 groups. For immunogram generation, *Z*-score normalization was applied for scores of aforementioned immune features, and the formula 3 + 1.5 × Z was used to further transform scores of each feature to depict the immunograms. Immunograms of the C1 group could then be compared to those of the C2 group. Moreover, all kinds of features were also used to explore differences between the groups using a boxplot and heatmap.

### 2.4. Statistical Tests and Prognosis MODEL Construction

Chi-square tests were used to determine whether there were differences in the problems of relapse within 1 year (yes vs. no) and the chemotherapy response (complete response (CR) vs. progressive disease (PD)) between groups C1 and C2. In addition, we utilized the sklearn (Python package, version v1.1.3) for machine learning and employed various classifiers, including random forest (RF), support vector machine (SVM), extreme gradient boosting (XGBoost), and adaptive boosting (AdaBoost), to make predictions. The Mann–Whitney U-test was applied to select discriminative features between the two groups from among 69 features. The RF parameters were set to n_estimator = 100 and criterion = gini, while SVM parameters were set to kernel = linear, degree = 3, and gamma = scale. XGBoost parameters were set to n_estimators = 3, and AdaBoost parameters were set to n_estimators = 50. Five-fold cross-validation was applied, and the accuracy, recall, precision, F1 score, and area under the curve (AUC) were used to evaluate model performances.

## 3. Results

### 3.1. Statistics of the Study Cohort

A total of 513 patients with lung adenocarcinoma (LUAD) were included in the study, of whom 177 had received chemotherapy. The demographic characteristics of the patients are presented in Table 1. The number of males and females in each group and the number of smokers are indicated in the table. In terms of population, the majority of subjects were identified as white, followed by African American. The mean age of all patients was 65.2 years, with 395 patients in the early-stage group (274 in stage I and 121 in stage II), 115 patients in the advanced-stage group (86 in stage III and 29 in stage IV), and 5 patients without this record. Among those who received chemotherapy, the mean age was 63.3, with 106 patients in the early-stage group and 68 patients in the advanced-stage group. Additionally, 62 and 29 patients receiving chemotherapy in the early-stage and the advanced-stage groups, respectively, exhibited a complete response (CR) to the drugs, while 25 and 17 demonstrated progressive disease (PD). Utilizing the mutation data available on the GDC portal, our findings revealed that 14–16% of patients exhibited EGFR mutations, while nearly 6% of patients demonstrated ALK mutations, both in all LUAD patients and in those who received chemotherapy.

### 3.2. Cell Proportions and Immunograms of Chemotherapy Patients

We sought to explore associations of cell proportions with the chemotherapeutic response. Cell proportions of all 513 LUAD patients and 177 chemotherapy patients are, respectively, shown in Figure S1A,B. Results showed that the highest average proportions were of basal cells and proliferative epithelial cells, and the lowest average proportions were of smooth muscle cells, mast cells, and endothelial cells among these samples. Moreover, to unravel the immune signatures among all patients and chemotherapy patients, immunograms were used and are provided in Figure S2. In both immunograms of all patients (Figure S2A) and chemotherapy patients specifically (Figure S2B), glycolysis and recognition of tumor cells exhibited the highest scores, while immune checkpoints and anti-CTLA4 resistance MAGE gene (CRMA) signature demonstrated the lowest. Furthermore, stromal scores exhibited a slight increase for chemotherapy patients in comparison to all patients.

### 3.3. PPS Score Evaluation of Patients with LUAD

Of 4617 immune-associated genes in InnateDB following the Cox regression and LASSO analyses, 23 genes were related to the prognosis of patients with LUAD (as shown in Table S2). Moreover, the PPS scores of patients were evaluated in depth with the LASSO Cox model, which was constructed by these 23 genes, and it was similar to a previous study [19]. The results demonstrated a significant divergence in prognoses between patients with high PPS scores and those with low PPS scores, which were divided by mean (as shown in Figure S3A,B). Patients with high PPS scores exhibited poor prognoses and were designated as a high-risk cohort, whereas those with low PPS scores demonstrated favorable prognoses and were classified as a low-risk cohort. Furthermore, the gene signatures of the two risk groups exhibited notable differences (as illustrated in Figure S3C).

### 3.4. Immune Profiles within Different Clusters

**Figure 2:**
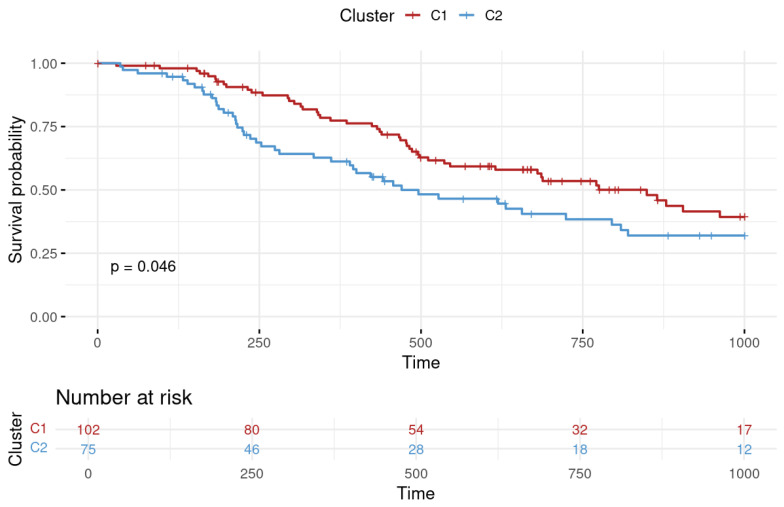
Progression-free interval (PFI) survival analysis for lung adenocarcinoma clusters. The survival probability over time is plotted with cluster 1 depicted in red and cluster 2 in blue. Time is represented on the *X*-axis in days, while the *Y*-axis indicates the survival probability. The number of patients at risk at various time points is provided below the graph, offering a detailed view of the cohort size over the study period.

In this study, we integrated 69 features including proportions of 17 cell types, 29 immune-relevant scores, and 23 PPS genes for the clustering of 177 patients who had undergone chemotherapy. Patients with LUAD and treated with chemotherapy were categorized into two clusters, as illustrated in the accompanying demographic table (Table S3). It was also notable that the PFI between the two clusters exhibited significant differences. Cluster C1 (*n* = 102) had a survival advantage compared to cluster C2 (*n* = 75). The *p* value of the log-rank test of survival durations of the two clusters was <0.05 (as shown in Figure 2).

**Figure 3:**
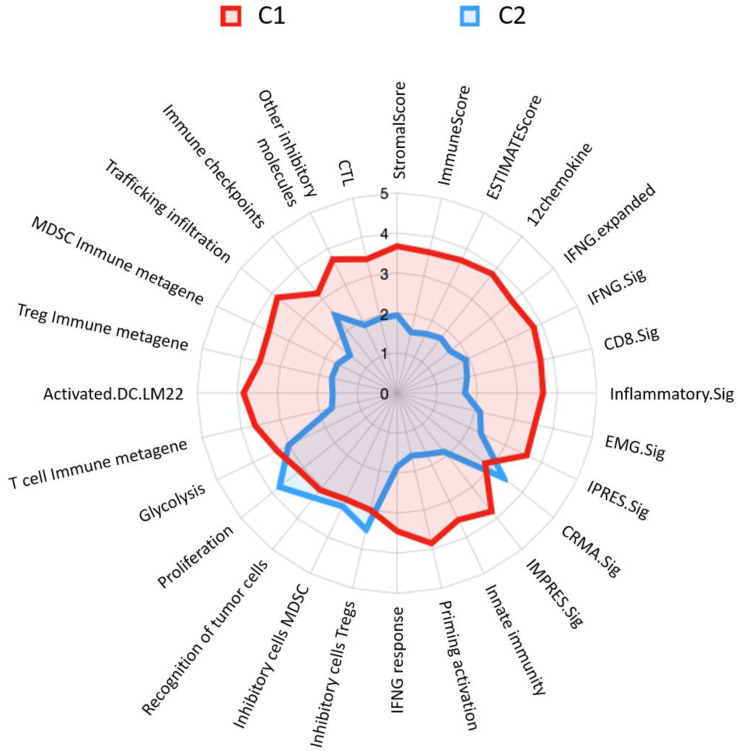
Immunoprofiling radar chart of lung adenocarcinoma patient clusters. C1 is represented by the red area with a good prognosis, while C2 is outlined in blue with a poor prognosis. Each axis on the radar represents a different immune signature. The values extend from the center of the radar chart outwards, with higher values indicating a more pronounced presence of the corresponding immune function within the cluster. Abbreviated information and a fully expanded response may be provided, as in Table S1.

On the other hand, we utilized immunograms to determine the difference in immunophenotypes between C1 and C2 (as shown in Figure 3). Several immune signatures differed such as CRMA signature, inhibitory cells Tregs, inhibitory cells MDSC, recognition of tumor cells, and proliferation, and were significantly higher in C2 than in C1. The CRMA and proliferation signatures obviously showed relatively large differences. Conversely, the remaining immune-relevant signatures were significantly higher in C1 than in C2. Among these signatures, trafficking infiltration and priming activation exhibited relatively large differences between the clusters.

**Figure 4:**
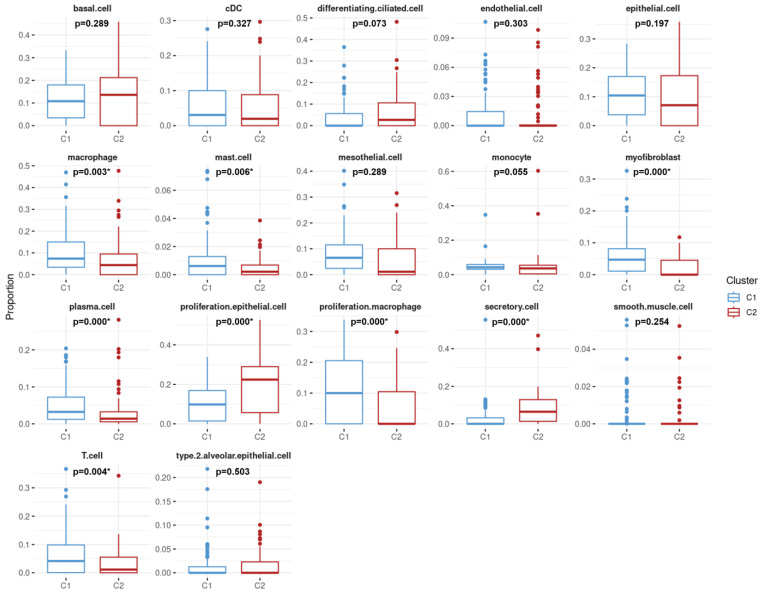
Comparative analysis of cell-type distribution between two lung adenocarcinoma patient clusters. C1 is represented by blue boxes, while C2 is shown in red. The *X*-axis categorizes the 17 cell types. The *Y*-axis quantifies the proportion of each cell type within the samples from each cluster. Statistical significance is indicated by *p*-values, with asterisks denoting *p*-values less than 0.05, suggesting significant differences in the distribution of certain cell types between the two clusters.

Moreover, distributions of cell proportions between the two clusters were further examined (as shown in Figure 4). Results demonstrated that the mean proportions of eight cell types significantly differed between the C1 and C2 clusters, including macrophages (0.10), mast cells (0.01), myofibroblasts (0.06), plasma cells (0.05), proliferative macrophages (0.10), and T cells (0.06), which were higher in C1 and proliferative epithelial cells (0.19) and secretory cells (0.09), which were conversely higher in C2.

**Figure 5:**
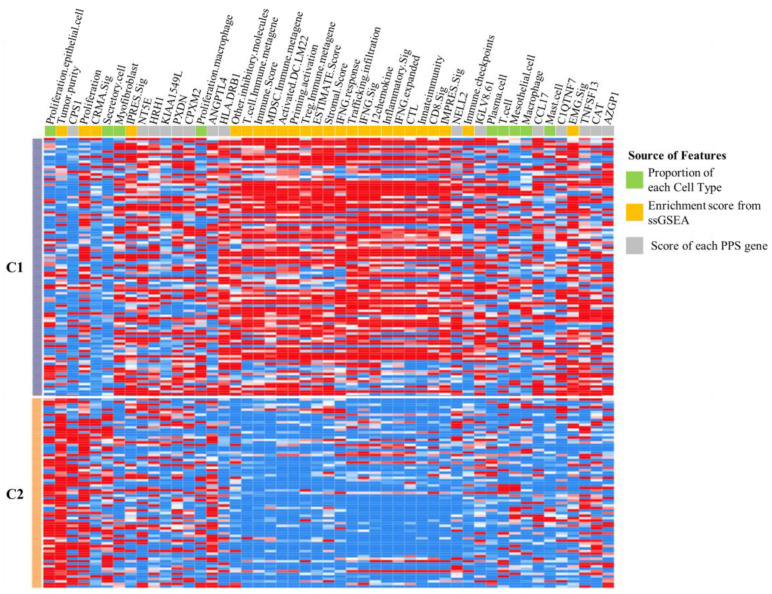
The heatmap depicts the two clustering groups of lung adenocarcinoma chemotherapy patients. The C1 group is represented by grey, while the C2 group is represented by orange. The features, including proportion of cell types and estimations of immune signatures via ssGSEA and PPS gene, are represented by green, yellow, and grey, respectively.

Among the 69 features in this study, 49 features exhibited statistically significant differences between clusters C1 and C2 (Table S4). Furthermore, the normalized source values of each feature, proportion of each cell type, enrichment score from ssGSEA, and score of each PPS gene were employed to generate a heatmap (as shown in Figure 5). The 9 features were derived from cell-type classification (designated in green), the 25 features were assessed by ssGSEA (designated in yellow), and the 15 PPS genes were designated in grey. Additionally, the majority of ssGSEA-assessed immune profiles exhibited a greater degree of enrichment in C1.

### 3.5. Correlation Analysis of Immune Clustering and Chemotherapeutic Response

According to the clustering results of patients who underwent chemotherapy, we further performed Chi-square tests with various scenarios between clusters. For disease recurrence within 1 year in these patients, the frequency statistically significantly differed between clusters C1 (20.5%) and C2 (36.0%), with a *p* value of 0.035 (Table 2).

Frequencies of the chemotherapy response among patients with LUAD also statistically significantly differed between clusters C1 and C2 (*p* = 0.0474). The CR in C1 at 75.6% was higher than that in C2 (59.6%), while PD in C1 at 24.3% was lower than that in C2 (40.3%) (Table 3).

### 3.6. Performance of Prognostic Models of Patients Who Underwent Chemotherapy

After exploring different clusters C1 and C2 of patients who had undergone chemotherapy, patients could potentially be divided into different risk groups with good or poor prognoses. Thus, the 49 critical features filtered in the previous section, “Immune profiles within different clusters”, were then used to construct prognostic models. Performances of the prediction models showed that the SVM had the highest accuracy of 0.955, while XGBoost had the lowest at 0.904. In terms of recall, SVM had the highest score of 1.000, while RF had the lowest at 0.931. Moreover, the highest precision was achieved by the SVM at 0.934, while XGBoost had the lowest precision at 0.898. For the F1 score, the SVM had the highest score at 0.964, while XGBoost had the lowest one at 0.918. Additionally, the SVM had the highest AUC score at 0.996, while RF and XGBoost had the lowest scores at 0.977. Detailed performances of the different models for each evaluation metric are shown in Table 4. These results demonstrated that the SVM outperformed other models and could be a promising tool for distinguishing good/poor prognoses among patients who had undergone chemotherapy.

## 4. Discussion

On the basis of several features, LUAD patients treated with chemotherapy could be divided into two different risk groups (C1 and C2). Relative to C2, higher levels of macrophages and T cells were expressed in C1 (as shown in Figure 4). Moreover, the immunogram demonstrated that the score of T cell immune metagene in C1 was higher than that in C2 (as shown in Figure 3). A previous study indicated that a high proportion of T cells was significantly associated with a better LUAD prognosis [25], while high infiltration of M1 macrophages was related to better overall survival of NSCLC patients [26]. Those studies were useful for describing reasons why both clusters had different risk profiles. On the other hand, cell-proportion-relevant features were derived from Bisque in this study. We also attempted to apply another tool, MuSiC, for cell decomposition, and it showed similar results to those of Bisque. For instance, in terms of the estimated proportion of plasma cells, Pearson’s correlation coefficient reached 0.98 between the MuSiC and Bisque tools.

In our immunogram analysis, we observed that the majority of immune-relevant features had higher scores in C1 (as shown in Figure 3). The features, including cytotoxic T lymphocytes (CTLs) and CD8 signature, were hypothesized to be associated with the prognosis of patients with LUAD, as a previous study indicated that high levels of CD8 T cells in the TME were associated with better prognoses [27]. On the other hand, myeloid-derived suppressor cells (MDSCs) were higher in C2; these cells were shown to play a key role in the immunosuppressive TME, and studies confirmed their involvement in the progression and prognosis of NSCLC [28]. In this study, different immunogram trends were correlated with patients’ prognoses (as shown in Figure 2 and Figure 3).

Liu et al. proposed the concepts of “hot” and “cold” tumors, with immune checkpoint inhibitors (ICIs) being ineffective against cold tumors that lack T cells [29]. Radiation and chemotherapy were found to induce changes in the TME of LUAD patients, with the detection of PD-L1 changing from negative to positive, indicating the transformation of cold tumors to hot tumors [30]. Our results showed that immune-associated features in cluster C1 were more highly activated and potentially belonged to hot tumors, with better prognoses and chemotherapy responses.

We utilized the LASSO Cox model to evaluate the PPS scores of patients with prognosis-associated genes. The aforementioned results showed that LUAD patients with lower PPS scores had better prognoses in terms of the PFI. We observed that among the filtered gene signatures, the *HLA.DRB1*, *NELL2*, *IGLV8.61*, *C1QTNF7*, *TNFSF13*, *CAT*, and *AZGP1* genes were highly expressed in cluster 1 and lowly expressed in cluster 2 (as shown in Figure S3C), which could be favorable factors for patient outcomes. Relative to cluster 1, however, the *CPS1* gene was highly expressed in cluster 2, which could be a poor factor for patient outcomes. According to previous reports, *HLA-DR* expression was positively correlated with T cell infiltration in the TME of LUAD patients [31]. Moreover, the *TNFSF13* gene was suggested to be a prognostic factor for NSCLC and a potential treatment target [28].

The constructed prediction model for C1 and C2 in this study was based on different kinds of features such as cell proportions, immune-relevant signatures, and PPS-associated genes. The SVM exhibited optimal performance in terms of accuracy (0.955), recall (1.000), and AUC (0.996) in predicting the good prognosis cluster (C2 in this study). This result is comparable to that of a high-performance model constructed by Chang et al., which employed the AdaBoost algorithm and achieved an accuracy of 0.962, a recall of 0.875, and an AUC of 0.938 in predicting a good prognosis [19]. The overall performance of the constructed model in this study indicated that our methods had good predictive ability for identifying good and poor prognoses of LUAD patients. In Chang et al.’s study, moreover, they focused on the relationships between the immune status and overall survival in patients with stage 3 or 4 NSCLC, whereas we investigated the impacts of the immune profiles of patients on the chemotherapeutic response and disease recurrence. The cell proportions and immune signatures identified in our study demonstrate robust correlations with disease prognosis, particularly in regard to progression-free survival (PFI). These findings indicate that specific immune cell populations, such as T cells and macrophages, along with immune signatures, can serve as reliable biomarkers for predicting patient prognosis. This illustrates the diagnostic potential of immune signatures and cellular compositions in LUAD, providing a foundation for future studies to utilize these biomarkers in clinical settings to assess patient outcomes with greater precision.

## 5. Conclusions

Given the complexity of the TME, we used the Bisque cell decomposition tool in combination with single-cell reference to estimate the cell proportions of LUAD patients. We integrated immune-related variables through the ssGSEA method to establish immunograms and identified immune-relevant genes, the expressions of which were significantly related to the PFI using the CoxPH model. Our findings suggested that cell proportions, immune signatures, and PPSs were associated with the chemotherapy response in LUAD patients. We recommend that future studies explore different cell annotation tools and leverage single-cell data to improve the accuracy of research results. Tools such as Bisque can improve the ability to disassemble cells in tumor tissues when data from bulk tumors are combined with single-cell data, and collecting single-cell data in clinical experiments may enhance research outcomes.

## Figures and Tables

**Table 1:** Demographic information of TCGA-LUAD samples.

Category		All (*N* = 513)	Chemo (*N* = 177)
Gender	Male	237	84
	Female	276	93
Smoker		351	116
Age (years)		65.2 ± 10.3	63.3 ± 9.9
Race	American Indian or Alaska Native	1	1
	Asian	7	4
	Black or African American	52	18
	White	387	138
Stage	I	274	37
	II	121	69
	III	86	53
	IV	29	15
Drug response (CR)	Stage I–II	--	62
	Stage III–IV	--	29
Drug response (PD)	Stage I–II	--	25
	Stage III–IV	--	17
With mutation	EGFR	73 (14.2%)	29 (16.4%)
	ALK	34 (6.6%)	11 (6.2%)

Abbreviations: TCGA-LUAD, The Cancer Genome Atlas lung adenocarcinoma; chemo, chemotherapy; CR, complete response; PD, progressive disease.

**Table 2:** Prognostic association analysis of clusters C1 and C2.

	PFI < 365 days	PFI ≥ 365 days	*p* Value
Cluster			0.035
C1 (*N* = 102)	21 (20.5%)	81 (79.4%)	
C2 (*N* = 75)	27 (36.0%)	48 (64.0%)	

PFI, progression-free interval.

**Table 3:** Drug response association analysis in clusters C1 and C2.

	CR	PD	*p* Value
Cluster			0.047
C1 (*N* = 78)	59 (75.6%)	19 (24.3%)	
C2 (*N* = 57)	34 (59.6%)	23 (40.3%)	

CR, complete response; PD, progressive disease.

**Table 4:** Evaluation of prediction accuracy of each model.

	RF	SVM	XGBoost	AdaBoost
Accuracy	0.910	0.955	0.904	0.921
Recall	0.931	1.000	0.940	0.951
Precision	0.919	0.934	0.898	0.920
F1 score	0.919	0.964	0.918	0.933
AUC	0.977	0.996	0.977	0.989

RF, random forest; SVM, support vector machine; XGBoost, extreme gradient boosting; AdaBoost, adaptive boosting; AUC, area under the curve.

## Data Availability

The data utilized in this study are publicly available. The RNA-seq data from lung adenocarcinoma patients were obtained from The Cancer Genome Atlas (TCGA), which can be accessed at https://portal.gdc.cancer.gov/ (accessed on 29 March 2021). Additionally, the single-cell sequencing datasets were retrieved from the Gene Expression Omnibus (GEO) under the accession numbers GSE148071, which are available at https://www.ncbi.nlm.nih.gov/geo/ (accessed on 24 January 2021).

## Supplementary Materials

The following supporting information can be downloaded at: https://www.mdpi.com/article/10.3390/cancers16183207/s1, Figure S1: the proportions of the 17 cell types; Figure S2: immunoprofiling radar chart of the lung adenocarcinoma patients; Figure S3: PPS score evaluation of patients with the lung adenocarcinoma. Table S1: the marker genes of 29 immune signatures; Table S2: 23 genes were related to the prognosis of patients with LUAD; Table S3: demographic information of two clusters of patients who received chemotherapy; Table S4: a differential analysis of the 69 features in the two cluster groups.
