# Supplementary material for: A Study of Disease Prognosis in Lung Adenocarcinoma Using Single-Cell Decomposition and Immune Signature Analysis
Source: Cancers (Basel). 2024 Sep 20;16(18):3207. doi: 10.3390/cancers16183207 (PMC11431002; doi:10.3390/cancers16183207)
Supplement: Supplementary file 1 [file cancers-16-03207-s001.zip › supplementary_figures_m.pdf]

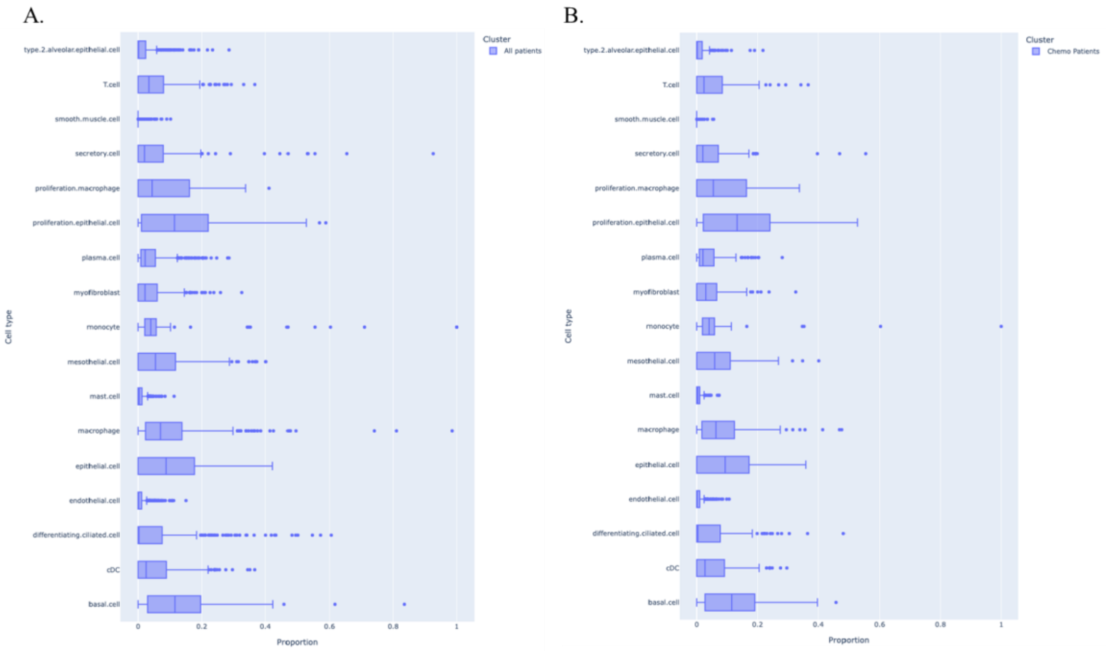

Figure S1. The proportions of the 17 cell types. (A) and (B) illustrate the cell proportions of all patients and those who received chemotherapy for lung adenocarcinoma, respectively.

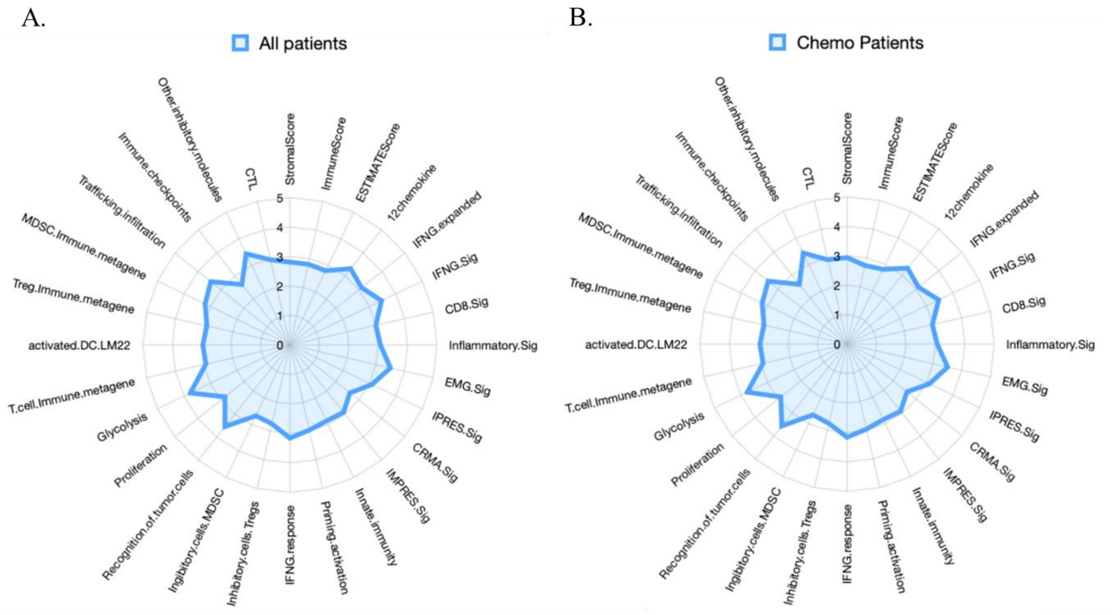

Figure S2. Immunoprofiling radar chart of the lung adenocarcinoma patients. (A) and (B) represent all patients and chemotherapy patients, respectively. The charts are plotted via ssGSEA for the purpose of evaluating the enrichment levels of different immune signatures.

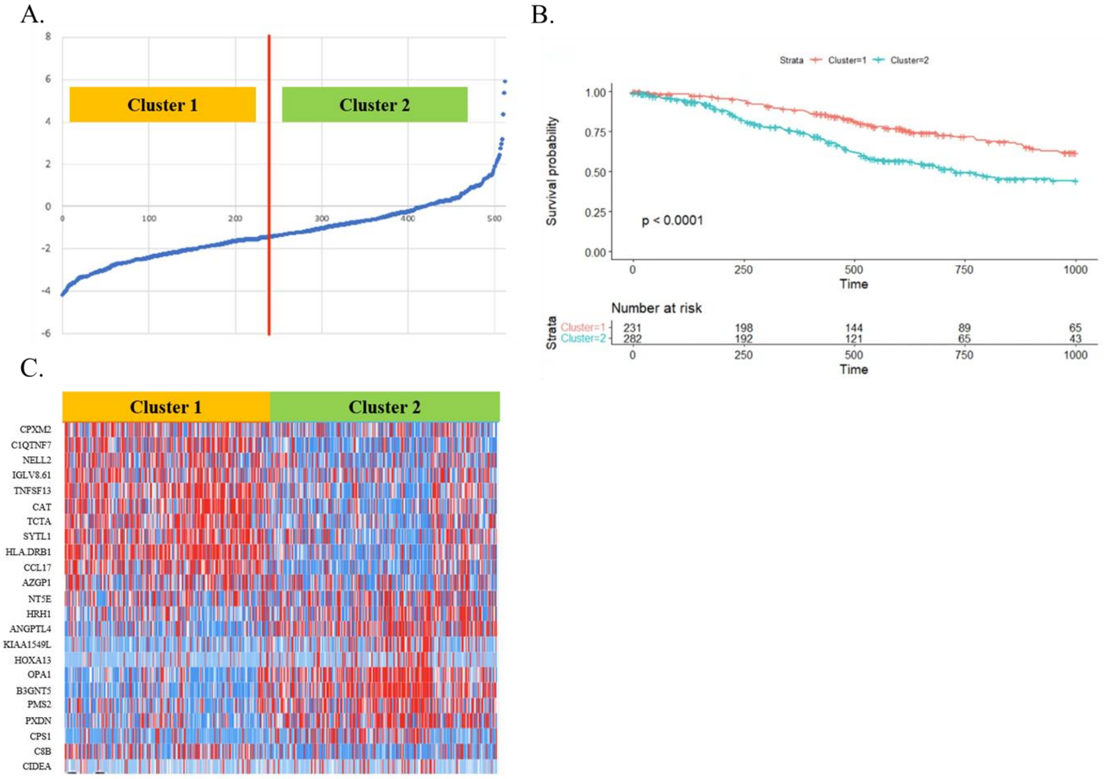

Figure S3. PPS score evaluation of patients with the lung adenocarcinoma. (A) The patients were divided into two clusters based on the mean PPS score. The low PPS score cluster is represented by yellow, while the high PPS score cluster is represented by green; (B) The survival analysis of the two clusters, with cluster 1 represented in red and cluster 2 in green; (C) The heatmap, which employs 23 PPS genes associated with prognosis.
